# Age- and Sex-Specific Reference Intervals for Myocardial Enzyme Activity in Healthy Chinese Han Population Aged 1∼<18 years

**DOI:** 10.1155/2019/2018598

**Published:** 2019-12-23

**Authors:** Wenjia Guo, Qi Zhou, Yanan Jia, Jiancheng Xu

**Affiliations:** ^1^Department of Laboratory Medicine, First Hospital of Jilin University, Changchun 130021, China; ^2^Department of Pediatrics, First Hospital of Jilin University, Changchun 130021, China; ^3^Shanxi Dayi Hospital, Taiyuan 030032, China

## Abstract

Age- and sex-specific reference intervals (RIs) for myocardial enzyme activity of children and adolescents are not available in China. Our study aimed to establish age- and gender-related RIs for AST, LDH, CK, and CKMB activity in healthy Chinese Han population aged 1∼<18 years. Healthy Han children and adolescents (*n* = 6322, 1∼<18 years old) were assessed from completed questionnaires and defined criteria from communities and schools in 5 administrative districts of Jilin Province from September 2017 to December 2018. Measurements of AST, LDH, CK, and CKMB activity were performed on the VITROS 5600 Integrated System. Percentiles of enzyme activity were completed by LMS. RIs were established by Medcalc according to the EP28-A3c guidelines issued by the Clinical and Laboratory Standards Institute. AST declined rapidly during 1∼<6 years and had been subsided during 11∼<18 years, though LDH decreased at a steady rate. CK activity stabilized while CKMB showed a downward trend. Sex differences started after age 12 when males presented higher results. There were significant differences comparing with domestic and other countries' experiments which applied similar methodologies. Enzymes were associated with age and sex, while age had greater impact. We established age- and sex-specific RIs of serum AST, LDH, CK, and CKMB activities for Chinese children and adolescents using the VITROS 5600 Integrated System for the first time. These data will lay the groundwork for the next horizon in pediatric RIs as well as improve test result interpretation for pediatric illness.

## 1. Introduction

Reference interval (RI) is a global research hotspot currently, referring to the range defined by 2.5 percentile and 97.5 percentile of a certain indicator among healthy population in most cases. As an effective tool, it provides valuable information for screening, diagnosis, treatment, and monitoring of diseases [1]. With the increase in public health awareness as well as precision and individualized medicine development nowadays, appropriate and accurate RIs are in great demand. Conversely, inappropriate RIs could lead to misdiagnosis and missed care, which hinder critical decision-making and lead to adverse consequences.

Research studies find [2–5] RIs are with variety of factors involved such as race, region, ethnicity, diet, and so on. For example, Wu et al. [6] found that lower limit of the platelet interval in Chinese Han adults was slightly lower than that of the peer group in the United States [7] and Malaysia [8], and RIs of white blood cell count and neutrophil count were lower than those of the homologous US population [7] while higher than those of the Africans [9]; Oliver's research [10] showed that RIs of certain biochemical indicators of the aged living in Asmara, Eritrea, were higher than those of Caucasians and lower than those of South Africans. For example, the actual upper limit of total bilirubin was 2 times higher than it was recommended by the Eritrean Ministry of Health; the median of creatine kinase (CK) activity of healthy black adults was about twice higher than Caucasians in both sexes [11]. Therefore, RIs establishment must be “adapted to local conditions,” or they cannot fully exert their effectiveness.

The Ministry of Health of China published “reference intervals for common clinical biochemistry tests” (WS/T 404-2012) for Chinese adults in 2012, while there are still few systematic pediatric RIs. Clinical practice has demonstrated that adult RIs were not suitable to assess pediatric health status and diseases [12], For instance, Lv [13] et al. stated that Chinese children's blood routine test RIs established by the direct method were not consistent with adults; Supriya [14] found acid *α*-glucose, glycosidase, acid *α*-galactosidase, acid *β*-glucocerebrosidase, acid sphingomyelinase, and galactocerebrosidase activity were different significantly among age groups in large-scale healthy Indian population; CALIPER (Canadian Laboratory Initiative on Pediatric Reference Intervals) and many scholars [15, 16] confirmed that transferrin saturation increased slightly with age. It was proved in our previous experiment that pediatric liver function indicators were quite different from adults [17]. All these indicate that it is quite essential to establish appropriate and practical pediatric RIs.

Aspartate aminotransferase (AST), lactate dehydrogenase (LDH), CK, and creatine kinase isoenzyme (CKMB) activity assays are recognized as effective indicators for cardiac function. Cardiomyocyte cytosolic enzymes release and increase significantly in serum when myocardial cells are damaged. AST is widely found in the myocardium, skeletal muscle, liver, and kidney, among which myocardial cells contain the most. LDH includes 5 isoenzymes: LDH_1_, LDH_2_, LDH_3_, LDH_4,_ and LDH_5_. LDH_1_ is mainly in the myocardium, accounting for more than 50% of total myocardial LDH activity. CK is mainly found in the skeletal muscle, brain, and myocardium. There are 4 isoenzymes such as CKMM, CKMB, CKMt, and CKBB. Most of the CKMB (95%) is located in the myocardial cytoplasm, being an important predictor of myocardial damage and correlating with infarct size positively [18, 19].

CK and CKMB increase instantly (3–8 h) as soon as the onset of acute myocardial infarction (AMI), peaking (10–36 h) and declining (72–80 h) in a relatively short time; AST and LDH start to ascend later (8–18 h), mount (24–72 h), and decline (6–10 d) slowly, being used to evaluate reinfarction, enlargement of infarction, recovery, and so on. These indicators make up the serum myocardial enzyme profile, being extremely important for diagnosis, treatment, and monitoring of cardiac diseases.

For example, researchers [20] found rotavirus infection, the most common cause of severe dehydration diarrhea in children under 5 years worldwide [21], led to high activity of CKMB [22] and CK [22, 23]; Xu and Shu [24] found serum AST and LDH of children with refractory pneumonia mycoplasma pneumonia appeared to be higher than those of children with general pneumonia mycoplasma pneumonia, and serum LDH was a crucial diagnostic clue for early diagnosis of refractory pneumonia mycoplasma pneumonia; Binder [25] claimed that AST/ALT of excessive-drinking adolescents differed from healthy peers by retrospectively investigating laboratory data, which reflected their hepatocyte damage.

Now, pediatric RIs of clinical laboratories in China vary in source, such as reagent instructions, textbooks, literature, and self-information databases. Data are outdated and mixed, and credibility and accuracy are doubtful [17]. We intend to establish feasible RIs of CK, CKMB, AST, and LDH activity in Chinese children and adolescents by recruiting a large number of healthy children in Jilin Province and promote the development of pediatric RIs project.

## 2. Materials and Methods

Healthy subjects were recruited from 5 administrative districts (including Changchun, Jilin, Baishan, Yanbian, and Songyuan) of Jilin Province. Subjects aged 1∼<18 years old, among children aged 1∼<7 years, were from the community and health centers, and children and adolescents aged 7∼<18 years were from the primary schools, junior middle schools, and high schools.

According to the EP28-A3c issued by the Clinical Laboratory Standard Institute (CLSI) [26], establishment of RIs requires collection of sufficient samples, i.e., no less than 120 individuals per partition. Due to the unknown interception point, we continued collecting samples from September 2017 to December 2018 to try for more than 120 children and adolescents at every year of age and each sex.

The experimenter first issued at designated institutions. Questionnaires include general questions such as height, weight, dietary, family history, surgical history, medication history, and so on. Exclusion criteria contained acute and chronic infections (upper respiratory tract infections, pneumonia, tuberculosis, syphilis, etc.), digestive diseases (cirrhosis, hepatitis, peptic ulcer, fatty liver, cholelithiasis, cholecystitis, chronic diarrhea, etc.), kidney diseases (chronic diseases, acute kidney injury, etc.), endocrine diseases (diabetes, metabolic syndrome, lipid and lipoprotein abnormalities, hyperuricemia, thyroid dysfunction, etc.), hematology diseases (anemia, leukemia, etc.), rheumatic diseases (rheumatoid arthritis, systemic lupus erythematosus, etc.), and cardiovascular diseases (hypertension-systolic blood pressure higher than 140 mmHg and/or diastolic blood pressure higher than 90 mmHg); no surgery was performed, no blood transfusion or donation within 4 months, and no medication was taken within 2 weeks recently.

Questionnaires were collected and revalued. Qualified subjects were assessed by a pediatrician on a fixed day. After the subjects were rescreened, blood was collected as required at the designated place on a fixed day every week. Laboratory exclusion criteria were as follows: HBsAg positive, HCV positive, or HIV antibody positive; serum creatinine >120 *μ*mol/L; serum uric acid >475 *μ*mol/L; fasting plasma glucose >7.0 mmol/L; or C-reactive protein >12.0 mg/L; serum creatine kinase >500 U/L.

The subjects were asked to maintain normal diet and exercise within 3 days and fasting overnight (>8 hours) before blood collection. Nurses were trained, and blood collection tubes were uniformly distributed (clot-activator tube containing gel, Vacutainer® SST, BD). Samples were collected and placed at room temperature for 30 minutes. After centrifuged at 3,000 rpm for 10 minutes, hemolysis, lipemia, or jaundice samples were removed. Specimens from non-Changchun were centrifuged and sent to the Central Laboratory (Department of Laboratory Medicine, First Hospital of Jilin University) within 8 hours; samples from Changchun were received and tested within 2 hours. The VITROS 5600 Integrated System (Johnson & Johnson) was applied to test serum AST (multipoint rate method; traceability of calibration to the aspartate aminotransferase method recommended by the International Federation of Clinical Chemistry (IFCC); adapted to a centrifugal analyzer at 37°C), LDH (multipoint rate method; traceability of calibration to the pyruvate-to-lactate (P ⟶ L) (Buhl) total lactate dehydrogenase method; adapted to a centrifugal analyzer at 37°C), CK (multipoint rate method; traceability of calibration to a modification of the Scandinavian Committee on Enzymes recommended method for the determination of creatine kinase at 37°C), and CKMB (multipoint rate method, traceability of calibration to a CK-M Immunoinhibition method with quantitation of the remaining CKB subunit activity by a modification of the Scandinavian Committee on Enzymes). Reagents and quality control products were purchased from Johnson & Johnson Company. The VITROS 5600 Integrated System underwent regular maintenance, function checks, calibration, quality control, and procedure according to the manufacturer's instructions. Analytical performance of the assays, such as precision, accuracy, analytical measurement range, and clinical reportable range, was monitored [17] and is shown in Table 1.

The Department of Laboratory Medicine at the First Hospital of Jilin University was accredited to ISO 15189:2012 Medical Laboratories-Particular Requirements for Quality and Competence by the China National Accreditation Service for Conformity Assessment (CNAS) in 2012. Clinicians, technicians, and nurses participating in the physical examination and sample analysis were trained and qualified. The VITROS 5600 Integrated System was regularly maintained, checked, calibrated, and quality controlled by the engineer and technicians.

**Figure 1:**
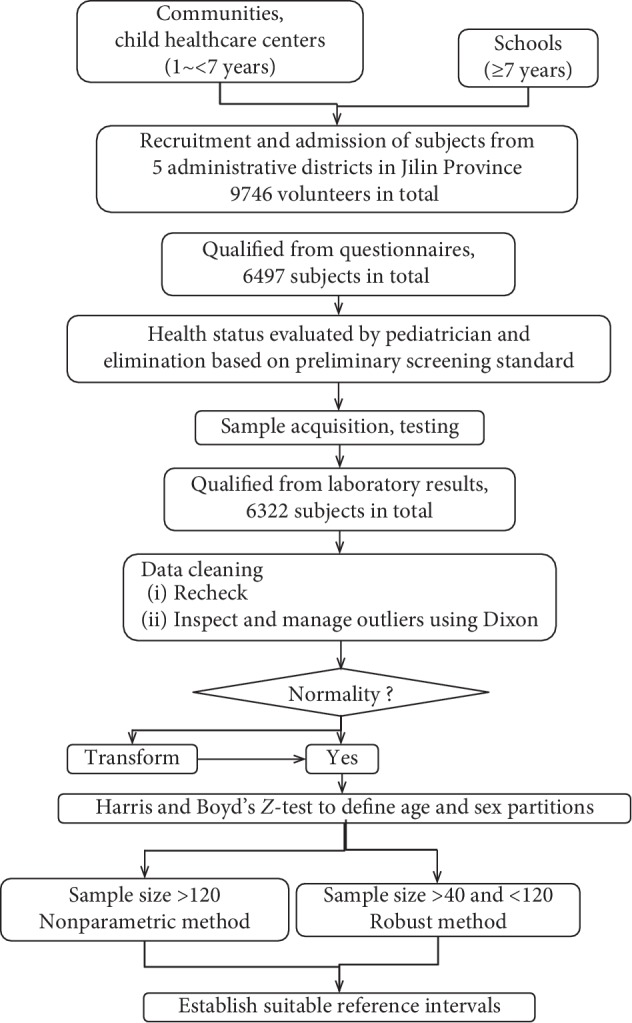
Protocol for establishment of pediatric reference intervals.

The database was rechecked. Cluttered ones from input errors, unknown sources, and blurred information were eliminated. Data processing was completed based on the EP28-A3c [26]. Outliers were deleted using the Dixon method; box plot was drawn to examine the results. Trends were assessed, and partitions were roughly determined by the scatter plot. Kolmogorov–Smirnov analysis was used to determine whether the subgroup followed Gaussian distribution (*P* < 0.05). Normal distributed subgroups were partitioned with Harris & Boyd's *Z*-test recommended by the CLSI, i.e., combination happened when *Z* exceeded *Z*^*∗*^; as for nonnormal distributed ones, the *Z*-test was conducted after Box–Cox transformation. After age and sex parturitions were finished, the nonparametric method was applied to calculate the lower and upper limits of RIs if the sample size was greater than 120; or RIs were calculated by the robust method when the number of cases was greater than 40 while less than 120. Referring to the EP28-A3c [26], the 90% confidence interval of limits was calculated. Details are summarized in Figure 1.

There were 9746 volunteers initially, while 6497 qualified after questionnaires. Laboratory results revealed that 6322 individuals met the inclusion criteria in total, among which 2998 subjects were from Baishan, 2193 ones from Changchun, 500 ones from Songyuan, 466 ones from Yanbian, and 165 ones were from Jilin (a city shared the same name). According to the final data, the nonparametric method was applied in calculation eventually.

Statistical analysis and figures were completed using LMS 2.54, Excel 2003, Medcalc 11.4.2.0, and SPSS 25.0. The LMS method was based on Box–Cox transformation, presenting 3 parameters, power (*λ*), median (*μ*), and coefficient of variation (*σ*) which were obtained by the maximum likelihood estimation method, and the effective degrees of freedom were adjusted to fit a smooth percentile curve with the age being the independent variable.

## 3. Results

### 3.1. Percentiles of Myocardial Enzyme Activity Index in Children

**Figure 2:**
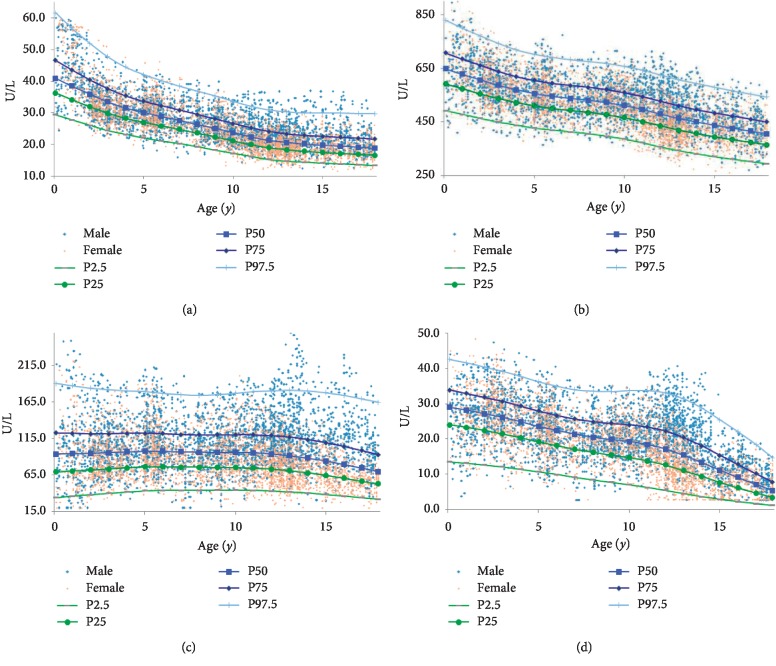
Age- and sex-specific scatter plots of (a) AST, (b) LDH, (c) CK, and (d) CKMB (1∼<18 years).

Eventually, 6,322 healthy children and adolescents (3,203 females and 3,119 males; male: female ratio 1 :1.03) were included.  Table 2 presents percentiles of AST, LDH, CK, and CKMB activity among the population aged 1∼<18 years. Combined with Figure 2, percentile 97.5, 75, 50, 25, and 2.5 of AST, LDH, and CKMB activity all showed downward trends and were associated with age significantly (*r* = −0.746, *P* < 0.01; *r* = −0.636, *P* < 0.01; *r* = −0.628, *P* < 0.01) while CK was relatively stable (*r* = −0.122, *P* < 0.01).

### 3.2. RIs Establishment of Myocardial Enzyme Activity

Considering the effects of exercise intensity on myocardial enzymes [27, 28], RIs were initially divided into 1∼<7 years, 7∼<12 years, 12∼<15 years, and 15∼<18 years. Age and sex partitions were determined by the *Z*-test, limits and confidence intervals were calculated (Table 3). Except for CK in males, the enzymes showed significant sex differences at the onset of age 7. Correlations of AST, LDH, CK, and CKMB with sex were positive (*r* = −0.145, *P* < 0.01; *r* = −0.157, *P* < 0.01; *r* = −0.356, *P* < 0.01; *r* = −0.2, *P* < 0.01).

AST declined rapidly in 1∼<6 years and had been subsided during 11∼<18 years; LDH decreased steadily; CK activity was quite stable. Males' results began to overwhelm their counterparts' at age 10. CKMB activity was a downward trend, with sex differences starting from age 12 (Figure 2). It is worth noting that 2.7 U/L is the detection limit; thus, the actual values might be lower.

### 3.3. Correlation between Myocardial Enzymes

There were strong correlations among indexes, and the coefficient between AST and LDH was *r* = 0.661, *P* < 0.01; the coefficient between AST and CK was *r* = 0.249, *P* < 0.01; the coefficient between AST and CKMB was *r* = 0.559, *P* < 0.01; the coefficient between LDH and CK was *r* = 0.361, *P* < 0.01; the coefficient between LDH and CKMB was *r* = 0.531, *P* < 0.01; the coefficient between CK and CKMB was *r* = 0.317, *P* < 0.01.

### 3.4. Comparison with Other Established Pediatric RIs

Table 4 lists similar experiments in other countries [3] and Guangdong (a southern city in China) [29] which applied similar detection systems and/or methodologies. Results showed that AST, LDH, and CKMB appeared with a decreased trend in all studies; other experiments stated that enzymes began to differ sexually from age 7∼<11, but sex partitions were not emphasized in Cao's experiment [29].

## 4. Discussion

### 4.1. Strengths

The VITROS 5600 Integrated System is mostly used in the emergency laboratory; thus, it is rarely used for establishing RIs in a large-scale population worldwide. CALIPER [32] tried to transfer the established RIs of Abbott ARCHITECHT to the VITROS 5600 Integrated System, while LDH and others did not meet the statistical criteria for transference. Therefore, advantages of this study are as follows: (1) reference population was continuously composited, and large sample size was completed (more than 6000 healthy subjects); (2) process of evaluation, screening, and testing were standardized; and (3) novel statistical and laboratory methods were applied.

### 4.2. Trends of Myocardial Enzyme

Enzymes were initially divided into 1∼<7 years, 7∼<12 years, 12∼<15 years, and 15∼<18 years, which were in agreement with Chinese students' in the primary, middle, and high school period. Compulsory sports and children's activities vary among these stages, and studies have shown that exercise intensity affects myocardial enzyme activity in a great way [27, 28]. From Figure 2 and Table 2, AST correlated with age obviously and males' results were higher, which was consistent with other scholars' results [33]. They believed AST related to puberty independent of age. Bussler considered that [33] the differences might be due to increased liver size, muscle mass, and fat-muscle distribution changing with age, which showed the most significant variation during adolescent [34, 35]. In addition, the interaction of hormones and metabolic changes with age (like insulin resistance) might lead to AST alternation between sexes in puberty [36–38].

Larsson [39] found that LDH activity was higher in young males compared with the elderly (*P* < 0.05); the activity of its muscle-type isoenzymes decreased with age (*P* < 0.05); cardiac-type isoenzyme activity increased though no statistical significance was observed. Therefore, it is suspected that decrease in LDH activity could be related to growing quantity of muscle mass with growth. Cao' research [29] applied the same detection system and methodology, and LDH declined to a greater extent while RIs were higher in the peer groups of Guangdong. It was presumed to be caused by climate, diet, and, so on.

Figure 2 displays that AST had the most dramatically descending rate in 1∼<6 years, followed by 6∼<11 years and being quite stable gradually till 11∼<18 years; males began to show higher level of AST than their counterparts at age 10. Percentiles of LDH activity were almost parallel and declined slightly with gentle fluctuation in 6∼<11 years, and sexual difference began at age 10 as well. The 97.5 percentiles of CK were extremely overexpectation, owing to discrepancy of muscle-type CK caused by various physical activities among individuals. Quite stable as CK activity, age 10 seemed to be a sexual difference watershed too. CKMB decreased at a constant speed, and sex differences occurred around 12 years when CKMB in males were obviously higher; a plateau appeared at 7∼<13 years of the 97.5 percentile, probably due to some high values in 11∼<14 years of male; the space between lines narrowed gradually, indicating stabilization. Correlation results proved that age-related coefficients of enzymes were higher than those with sex, and it was known that age had a greater impact.

Except CK in male, the others showed significant differences in sex after age 7. It could be due to more exercise as well as muscle mass in boys with growth [29]. After puberty, males express a strong interest in sports. Besides, physical education systems in primary, middle, and high schools in Changchun might cast different physical fitness.

CKMB is the most discrepant indicator compared with Cao's results [29], mainly in sex partition. In other trials, myocardial enzyme activity of adolescents was similar to that of adults after age 12, even lower than the detection limit. Exercise tests supposed that CKMB rose after exercise was not cardiac but muscle sourced (skeletal muscle contained about 7% CKMB) [27]; thus, exercise mainly affected CK but not CKMB. We have not found relevant logical physiological explanation.

### 4.3. Comparison of RIs with Other Similar Studies

The VITROS 5600 Integrated System was applied in our experiment, and there was no such trial to establish RIs of myocardial enzyme activity in large-scale healthy Chinese and even Asian healthy young generation at present. CALIPER [32] had stated that RIs of LDH activity could not be transferred to the VITROS 5600 Integrated System. Therefore, our study established RIs of myocardial enzyme activity in Chinese children for the first time. Comparing the research using the same systems and methodologies, we found that the lower limit of the AST interval was similar to that of children in Guangdong [29] while the upper limit was much lower, especially after age 12. RIs in other experiments were obviously higher than those of Chinese, which could be caused by ethnicity. The lower limit of LDH was lower than Cao's [29] with gap gradually decreasing with age while the upper limit was much higher. Just as CK, i.e., the lower limits were similar but the upper limit was higher in Guangdong subjects. CKMB owned the opposite situation: lower limit in Guangdong was 7 U/L, while the lower limit of females aged 12 in this experiment was 2.4 U/L and 15∼<18 year older ones' results have reached the lower limit of 2.7 U/L. Actual values might be lower. It is speculated that the basal metabolic rate was higher as a consequence of hot weather in Guangdong, and enzyme activity was also affected. It is difficult to compare CKMB activity with other experiments [40] due to different methodologies, while the lower limit had reached the detection limit likewise. Cao et al [29] did not partition AST, LDH, and CKMB based on sex, with RIs ranged broad, and it was different from our experiment.

RIs of LDH activity were higher than those of Chinese healthy adults (120∼250 U/L, rate method). RIs of CK were significantly lower than those of healthy adults (male 50∼310 U/L, female 40∼200 U/L, rate method). The CK results of Cao's [29] were more consistent with the adults' results. It is speculated that the muscles were still immature, mingling with changes of CKMM. AST in this experiment was quite consistent with RIs of adults (male 15∼40 U/L, female 13∼35 U/L) and in adolescents.

### 4.4. Limitations

Disadvantages are as follows: (1) the 97.5 percentiles of CK and CKMB were quite over but evenly distributed, probably due to participants' intense physical activity; (2) analysis of how factors such as diet, exercise intensity, climate, and so on affect should be explored in the future; and (3) more results with similar systems and methodologies of other regions or ethnics should be compared with.

## 5. Conclusion

Underestimation or overestimation of clinical measurements can lead to missed/misdiagnosed and expensive and unnecessary medical follow-up. For RIs being most effective, a reliable and accurate process of establishment should include appropriate parameters such as age, sex and ethnicity, subject health, local population, analytical methods, and so on. However, establishing a reference interval consumes much time, labor, and money.

RIs could vary among domestic studies and abroad ones with the same methodologies. Therefore, it is essential to establish a suitable and practical database. These data will lay the groundwork for the next horizon in pediatric RIs as well as improve test result interpretation for pediatric patients.

## Figures and Tables

**Table 1:** Analytic parameters of myocardial enzyme activity on the VITROS 5600 Integrated System.

Enzyme	SI unit	Bias of accuracy (%)	Precision				Analytical measurement range (U/L)	Clinical reportable range (U/L)	Accreditation criteria	
			Low level (%)		High level (%)							
			Within-day	Between-day	Within-day	Between-day			Bias^*∗*^ (%)	TE^*∗*^ (%)
AST	U/L	4.86	1.40	1.42	1.40	1.52	9.0–653.0	9.0–6530.0	5.00	15.00
LDH	U/L	1.91	2.30	2.33	1.85	1.90	294.0–1473.0	294.0–14730.0	4.00	11.00
CK	U/L	4.85	2.15	4.20	1.79	3.27	21.5–1566.0	21.5–1566.0	5.50	15.50
CKMB	U/L	5.51	5.18	5.20	4.30	4.31	2.7–300.0	2.7–300.0	7.00^*∗*^	22.00^*∗*^

^*∗*^TE refers to total error; Bias and TE criteria are from *Analytical quality specification for routine analytes in clinical biochemistry* published by the Ministry of Health of the People's Republic of China; due to lack of criteria for CKMB, bias and TE were recommended by our own clinical laboratory. Coefficient of variation (CV) for within-day precision should be < 1/4TE, and CV for between-day precision should be < 1/3TE according to *Guidance on the Application of Accreditation Criteria for the Medical Laboratory Quality and Competence in the Field of Clinical Chemistry* published by the China National Accreditation Service for Conformity Assessment.

**Table 2:** Percentiles of myocardial enzyme activity indicators of Chinese Han children (*n* = 6322).

Age (year)	*n*	*n,* M/F	Height (cm), M/F	Weight (kg), M/F	BMI (cm/kg^2^), M/F	AST (U/L)					LDH (U/L)					CK (U/L)					CKMB (U/L)				
						*P*_2.5_	*P*_25_	*P*_50_	*P*_75_	*P*_97.5_	*P*_2.5_	*P*_25_	*P*_50_	*P*_75_	*P*_97.5_	*P*_2.5_	*P*_25_	*P*_50_	*P*_75_	*P*_97.5_	*P*_2.5_	*P*_25_	*P*_50_	*P*_75_	*P*_97.5_
1∼<2	263	128/135	83.8/83.2	11.0/10.9	15.7/15.7	29.1	36.2	41.6	50.6	57.2	458.8	586.0	641.0	696.0	829.0	24.3	60.5	92.6	125.8	220.1	11.6	22.7	27.0	31.4	41.1
2∼<3	468	231/237	95.1/93.8	14.4/13.8	15.8/15.7	24.8	29.9	33.1	36.2	44.6	459.7	545.0	592.0	642.0	732.5	46.2	71.0	94.2	112.7	187.4	15.2	23.4	28.0	32.5	41.5
3∼<4	384	191/193	102.0/100.0	16.3/15.7	15.7/15.6	24.7	28.2	31.2	34.0	41.1	450.0	533.0	577.0	613.0	712.4	46.5	75.6	93.7	114.9	173.4	10.6	20.4	25.5	30.5	39.0
4∼<5	247	123/124	110.0/106.9	19.1/17.9	15.7/15.6	22.1	26.9	29.8	33.0	39.9	416.8	502.0	546.0	600.0	676.6	47.5	75.5	94.8	121.5	185.7	12.2	20.2	24.5	28.8	37.0
5∼<6	614	297/317	117.4/115.9	21.3/20.6	15.4/15.3	22.2	27.2	29.8	33.1	40.0	424.4	505.0	552.0	601.3	705.4	54.0	81.8	102.5	126.7	187.4	9.6	19.0	23.2	26.7	35.1
6∼<7	243	122/121	122.9/121.7	24.0/23.2	15.8/15.7	20.3	25.3	27.9	32.2	39.3	419.2	490.0	528.0	581.0	680.3	42.2	77.7	95.1	112.3	170.8	10.2	16.8	20.7	25.0	34.0
7∼<8	243	123/120	128.6/128.8	25.8/25.8	15.5/15.5	19.7	25.0	27.4	32.1	39.1	421.1	484.0	522.0	580.0	664.3	29.6	68.8	88.2	115.8	161.8	2.7	14.9	19.5	23.2	32.3
8∼<9	283	162/121	136.8/133.6	30.4/28.8	16.2/16.1	19.3	23.3	25.8	28.6	36.6	419.5	495.0	532.0	569.0	652.9	43.8	76.2	93.6	115.7	163.4	7.5	16.8	20.3	24.1	31.8
9∼<10	446	224/222	142.9/142.6	33.6/33.2	16.4/16.2	18.3	22.1	24.8	28.5	34.5	405.5	481.0	532.0	574.3	653.6	38.8	75.7	98.3	115.7	170.6	9.3	16.8	20.3	23.9	32.3
10∼<11	398	207/191	149.9/150.9	39.8/39.7	17.6/17.4	16.1	20.4	22.9	26.5	34.1	381.0	477.8	518.0	567.3	655.0	44.9	75.7	100.4	124.6	184.2	8.9	16.2	19.2	22.8	31.5
11∼<12	436	192/244	158.7/157.7	45.4/45.4	18.0/18.2	15.5	19.2	21.3	24.0	32.8	353.9	440.0	484.0	537.0	647.3	47.7	75.2	96.7	118.3	166.6	6.2	14.9	19.3	24.5	34.7
12∼<13	715	362/353	164.4/161.0	51.1/48.4	18.9/18.6	15.0	18.3	20.5	23.5	30.4	341.9	416.0	462.0	522.0	640.2	43.0	73.5	92.4	118.2	186.5	4.4	11.9	17.7	23.3	35.0
13∼<14	507	250/257	169.8/161.9	55.3/50.9	19.1/19.4	14.1	18.0	20.3	22.7	29.7	327.7	401.0	446.0	491.0	588.0	41.2	67.5	87.9	118.8	225.0	3.3	9.9	14.3	19.9	31.0
14∼<15	246	122/124	171.1/163.2	57.8/54.1	19.7/20.3	14.7	17.8	20.2	22.6	33.8	335.1	410.3	462.0	500.0	600.0	38.8	68.0	88.6	109.8	176.5	2.7	6.9	9.8	14.9	29.6
15∼<16	330	132/198	175.0/163.7	62.8/53.8	20.5/20.1	13.9	17.6	19.8	22.3	30.8	314.8	392.0	431.0	464.5	558.7	34.7	60.6	79.4	103.1	176.2	2.7	5.0	8.0	11.3	19.2
16∼<17	247	124/123	176.5/164.3	64.4/54.1	20.7/20.0	13.9	17.4	19.7	22.9	33.5	305.2	380.0	427.0	475.0	564.8	38.8	58.0	78.0	117.5	205.1	2.7	7.8	10.5	13.6	19.7
17∼<18	252	129/123	180.0/164.2	67.9/55.4	20.9/20.5	13.1	16.6	18.8	21.8	27.1	306.6	371.8	409.0	457.0	543.0	31.8	53.3	71.3	94.2	152.1	2.7	2.7	5.9	8.6	14.4

**Table 3:** Sex- and age-specific reference intervals for myocardial enzyme activity in healthy population aged 1∼<18 years (*n* = 6322).

Analytes	Age group	Sex group	No. of samples	Lower limit	Upper limit	Confidence interval for lower limit	Confidence interval for upper limit
AST (U/L)	1∼<7	M + F	2219	22.7	51.9	22.3–23.0	50.6–52.9
	7∼<12	M	908	18.2	36.4	17.4–18.6	35.5–37.1
		F	898	16.0	33.8	15.3–16.3	33.6–34.9
	12∼<18	M	1119	15.4	33.3	15.0–15.8	32.3–33.8
		F	1178	13.7	26.6	13.2–14.1	26.3–27.3

LDH (U/L)	1∼<7	M + F	2219	434.9	737.7	431.2–438.6	732–743.4
	7∼<12	M	908	408.8	663.7	402.9–414.7	657.3–670.1
		F	898	373.8	642.1	367.9–379.8	635.0–649.1
	12∼<15	M	734	364.8	641.0	359.1–370.5	631.7–650.4
	15∼<18	M	385	314.6	568.6	305.9–323.3	558.6–578.7
	12∼<18	F	1178	315.7	554.9	311.6–319.6	548.6–561.2

CK (U/L)	1∼<18	M	3119	49.1	194.1	47.1–50.9	191.3–197.7
	1∼<7	F	1127	41.0	166.5	39.4–42.7	162.6–170.5
	7∼<12	F	898	39.3	155.4	37.5–41.2	151.6–159.2
	12∼<15	F	734	36.6	129.2	35.0–38.2	125.8–132.7
	15∼<18	F	444	32.0	105.9	30.4–33.6	102.3–109.6

CKMB (U/L)	1∼<7	M + F	2219	11.4	38.9	11.0–11.8	38.4–39.3
	7∼<15	M	1642	7.9	34.2	7.5–8.4	33.7–34.7
	7∼<12	F	898	7.4	30.7	6.8–8.0	30.2–31.3
	12∼<15	F	734	2.8	23.3	2.5–3.1	22.5–24.1
	15∼<18	F	444	2.7^*∗*^	15.5	2.7^*∗*^	14.5–16.9
	15∼<18	M	385	2.1	20.8	1.7–2.5	19.9–21.8

^*∗*^2.7 U/L is the lowest detection limit. Actual values could be lower. M + F indicates that RI is shared by both genders.

**Table 4:** Comparison of reference intervals for myocardial enzyme activity applied with similar detection systems and/or methodologies.

	This study				Other country research				Cao et al. [29]			
	System/method	Age	Sex	RI	System/method	Age	Sex	RI	System/method	Age	Sex	RI
AST (U/L)	VITROS 5600/multipoint rate	1∼<7	M + F	22.7–51.9	VITROS 5600/rate [30]	1∼<7	M + F	33–66	VITROS 350/rate	3 m∼<3 y	M + F	23–60
		7∼<12	M	18.2–36.4		7∼<12	M + F	28–50		3 y∼<12 y	M + F	24–47
			F	16.0–33.8		12∼<18	M	24–49		12∼<18	M + F	17–47
		12∼<18	M	15.4–33.3			F	23–41				
			F	13.7–26.6								

LDH (U/L)	VITROS 5600/multipoint rate	1∼<7	M + F	434.9–737.7	Abbott ARCHITECT/rate [30]	1∼<10	M + F	309–1222	VITROS 350/rate	3 m∼<3 y	M + F	504–896
		7∼<12	M	408.8–663.7		10∼<15	M + F	163–452		3 y∼<12 y	M + F	491–790
			F	373.8–642.1		15∼<19	M + F	192–321		12∼<18	M + F	342–716
		12∼<15	M	364.8–641.0								
		15∼<18	M	314.6–568.6								
		12∼<18	F	315.7–554.9								

CK (U/L)	VITROS 5600/multipoint rate	1∼<18	M	49.1–194.1	VITROS/rate [31]	13∼24 m	M	27–160	VITROS 350/rate	3 m∼<12 y	M + F	50–226
		1∼<7	F	41.0–166.5			F	24–175		12 y∼<18 y	M	47–256
		7∼<12	F	39.3–155.4		2∼14	M	30–150			F	34–235
		12∼<15	F	36.6–129.2		2∼10	F	24–175				
		15∼<18	F	32.0–105.9		11∼14	F	30–170				
						15∼18	M	33–145				
							F	27–140				

CKMB (U/L)	VITROS 5600/multipoint rate	1∼<7	M + F	11.4–38.9					VITROS 350/rate	3 m∼<6 y	M + F	18–44
		7∼<15	M	7.9–34.2						6 y∼<12 y	M + F	7–35
		7∼<12	F	7.4–30.7						12 y∼<18 y	M + F	7–28
		12∼<15	F	2.8–23.3								
		15∼<18	F	2.7^*∗*^–15.5								
		15∼<18	M	2.1–20.8								

^*∗*^Lowest detection limit. Actual values could be lower.

## Data Availability

The datasets used and/or analysed during the current study are available from the corresponding author on reasonable request.
